# The Genetic Association of Variants in *CD6*, *TNFRSF1A* and *IRF8* to Multiple Sclerosis: A Multicenter Case-Control Study

**DOI:** 10.1371/journal.pone.0018813

**Published:** 2011-04-28

**Authors:** The International Multiple Sclerosis Genetics Consortium

**Affiliations:** Université Paris Descartes, France

## Abstract

**Background:**

In the recently published meta-analysis of multiple sclerosis genome-wide association studies De Jager et al. identified three single nucleotide polymorphisms associated to MS: rs17824933 (*CD6*), rs1800693 (*TNFRSF1A*) and rs17445836 (61.5 kb from *IRF8*). To refine our understanding of these associations we sought to replicate these findings in a large more extensive independent sample set of 11 populations of European origin.

**Principal Findings:**

We calculated individual and combined associations using a meta-analysis method by Kazeem and Farral (2005). We confirmed the association of rs1800693 in *TNFRSF1A* (p 4.19×10−7, OR 1.12, 7,665 cases, 8,051 controls) and rs17445836 near *IRF8* (p 5.35×10−10, OR 0.84, 6,895 cases, 7,580 controls and 596 case-parent trios) The SNP rs17824933 in *CD6* also showed nominally significant evidence for association (p 2.19×10−5, OR 1.11, 8,047 cases, 9,174 controls, 604 case-parent trios).

**Conclusions:**

Variants in *TNFRSF1A* and in the vicinity of *IRF8* were confirmed to be associated in these independent cohorts, which supports the role of these loci in etiology of multiple sclerosis. The variant in *CD6* reached genome-wide significance after combining the data with the original meta-analysis. Fine mapping is required to identify the predisposing variants in the loci and future functional studies will refine their molecular role in MS pathogenesis.

## Introduction

Multiple sclerosis (MS) is a complex neurological autoimmune disease with few known predisposing factors. Both genetic and environmental components have been predicted to play a role in MS etiology and the role of the *HLA*-locus, *HLA-DBR1* in particular, is well recognized [1], [2]. Recently, genome-wide association and candidate gene studies have revealed significant associations to MS outside the *HLA*-locus in *IL2RA* [2], *IL7R* [2], *CD58* [3], *CLEC16A* [4], *TYK2* [5], *STAT3* [6], *IL12A*, *MPHOSPH9/CDJ2AP1*, *EVI5* [2], *KIF21B* [2], [7], *TMEM39A* [2], [7], *CYP27B1* [8], *CD226* [4], *CD40* [8], *CBLB* [9] and *RGS1* [10], but with modest odds ratios suggesting the involvement of other loci.

In a recently published meta-analysis of six genome-wide analysis (GWA) study sets of 2,624 MS cases and 7,220 controls from four populations of European origin (United States, United Kingdom, Netherlands and Switzerland), De Jager et al. identified three single nucleotide polymorphisms (SNPs) associated with MS with significance exceeding the genome-wide significance level of p<5×10^−8^: rs1800693 in *TNFRSF1A*, rs17445836 61.5 kb from *IRF8* and rs17824933 in *CD6* [11]. De Jager et al. replicated these findings in 2,215 cases and 2,116 controls from UK and US. Recently, there have been reports showing significant genetic differences in allele frequencies between populations even within Europe [12], [13], [14] which has led to speculation of allelic heterogeneity. We set out to replicate the association of these SNPs to MS in a more extensive sample set with varying European origins.

## Results

**Figure 1:**
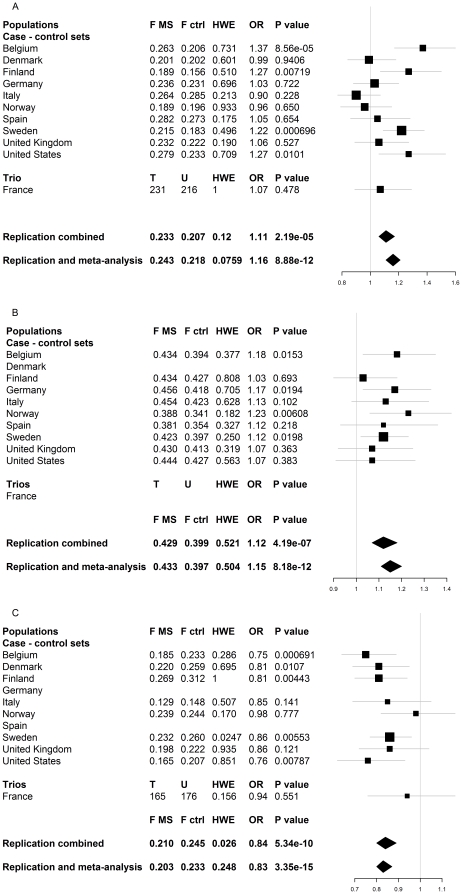
Summary of results. The results for individual populations are presented here each population on its own line. For each population we report the allele frequency in MS patients (F MS) and controls (F ctrl), Hardy-Weinberg (dis)equilibrium (HWE) p value, odds ratio (OR) and association p value. The association analyses were performed according to Kazeem and Farral [15]. The reported HWE p value is reported for cases and controls combined, but no significant deviation was observed within cases or controls when analyzed separately (data not shown). Figure 1a represents the results for rs17824933 in *CD6*. The Replication -line is the combined result of all independent sample sets in the replication (8,047 cases, 9,174 controls, 604 case-parent trios) and “Combined with De Jager et al. GWA” set includes the De Jager et al. [11] GWA data set (2,624 cases, 7,220 controls). Figure 1b summaries the results for rs1800693 in *TNFRSF1A*. Genotyping was unsuccessful in two sample sets (Danish case – control set and French case-parent trios) for rs1800693. Indipendent replication data set (“Replication”) included total of 7,665 cases and 8,051 controls and the “Combined with De Jager et al. GWA” set includes available genotypes from De Jager et al. [11] (1,829 cases, 2,591 controls). Figure 1c is a summary of results for rs17445836 (61.5 kb from *IRF8*). The genotyping was unsuccessful in two sample sets (Spanish and German case – control sets). The independent replication set (Replication) includes in total 6,895 cases, 7,580 controls and 596 case-parent trios and the “Combined with De Jager et al. GWA” set includes available genotypes from De Jager et al. [11] (2,624 cases, 7,220 controls).

We investigated the top three SNP associations by De Jager et al. (rs1800693 in *TNFRSF1A*, rs17445836 61.5 kb from *IRF8* and rs17824933 in *CD6*) in an independent sample set of 11 populations of varying European origins, comprising a total of 8,439 cases, 9,280 controls and 608 case-parent trios (Table 1). Cases and controls were selected from the same populations to minimize population stratification. We performed meta-analysis using a method by Kazeem and Farrall (2005) [15] and observed nominal association (p<0.05) with multiple sclerosis for rs17824933 in *CD6* in four of the eleven cohorts (Figure 1a), for rs1800693 in *TNFRSF1A* in four out of nine available cohorts (Figure 1b) and for rs17445836 near *IRF8* in five out of nine available cohorts (Figure 1c) (see materials and methods for details).

**Table 1:** Summary of all independent replication sample sets.

Sets	N trios	N ctrl	N MS	% PPMS	Sex ratios F∶MMS, ctrl	EDSS	disease duration	Genotyping platform
Belgium	0	1,021	776	13.7	1.8∶1, 1.1∶1	4.8	14	TaqMan® (Applied Biosystems)
Denmark	0	1,090	634	7.6	2.0∶1, 1.6∶1	4.1	12	Sequenom® iPLEX® Gold
Finland	0	1,077	792	9.4	2.4∶1, 1.4∶1	4.5	21	Sequenom®,TaqMan®*
France	608	0	0	12.0	2.4∶1, 1.0∶1	3.4	9.1	TaqMan® (Applied Biosystems)
Germany	0	911	930	<1%	n.a.	n.a.	7	Sequenom® iPLEX® Gold
Italy	0	629	828	11.1	2.0∶1, 1.0∶1	3.2	32	TaqMan® (Applied Biosystems)
Norway	0	1,027	662	17.7	2.6∶1, 2.0∶1	4.6	16	Sequenom®,TaqMan®*
Spain	0	501	501	19.9	1.8∶1, 1.1∶1	4.2	14	TaqMan® (Applied Biosystems)
Sweden	0	1,723	2,016	5.8	2.5∶1, 2.0∶1	3.3	n.a.	Sequenom® iPLEX® Gold
United Kingdom	0	714	656	14.4	2.8∶1, 2.8∶1	4.8	18	Sequenom® iPLEX® Gold
United States	0	587	644	12.0	1.1∶1, 1.1;1	4.1	15	Sequenom® iPLEX® Gold
Total	608	9,280	8,439		2.1∶1, 1.4∶1			

All sample sets for the replication are independent, cases had clinically definite MS by either the Poser or McDonald criteria and anonymous population samples from respective populations were used as controls. The clinical parameters for MS patients describe the percentage of primary progressive MS (PPMS) of all cases, the mean EDSS score and the mean disease duration. The original GWA meta-analysis sample sets by De Jager et al. that were used in the combined analysis of the original GWA results and our independent replication have been described elsewhere [11], [16].

*The Norwegian and Finnish samples were genotyped with the Applied Biosystems TaqMan® platform for rs1800693 and Sequenom® iPLEX® Gold for rs17624933 and rs17445836.

In all except three cohorts (Denmark, Italy and Norway for the *CD6* rs17824933 C allele) allele frequency differences between cases and controls had a trend towards the same direction as seen in the original meta-analysis [11] (Figure 1).Most of the individual cohorts had limited estimated power (varying between 25–82%, alpha 0.05) to observe the association by themselves (Table S1). Nevertheless, the estimated power for a combined analysis was >99% (alpha 0.05) to detect association to variants with the same effect sizes as observed in the original meta-analysis (rs1800693 OR 1.2, rs17445836 OR 0.80, rs17824933 OR 1.18).

The combined analysis confirmed independent associations with two of the SNPs with odds ratios comparable to those observed in the original meta-analysis: rs1800693 in *TNFRSF1A* (p 4.19×10^−7^, OR 1.12, 95% CI 1.07–1.18) and rs17445836 near *IRF8* (p 5.34×10^−10^, OR 0.84, 95% CI 0.80–0.89) (Figure 1b and c, respectively). Nominally significant association for rs17824933 in *CD6* was also observed (p 2.19×10^−5^, OR 1.11, 95% CI 1.06–1.17) (Figure 1a). Combining the replication data with the original meta-analysis data from De Jager et al. did not significantly change the observed odds ratios (Figure 1). We noticed an unequal distribution of minor allele frequencies across European populations as might be expected [12], [13], [14] in the rs17445836 and rs17824933 SNPs (Figure 1). However, the Breslow-Day test confirmed that there was no major heterogeneity in the odds ratios, although the allele frequency differences were significant between several populations when controls from different populations were compared in a pair-wise manner with a standard association tests (Table S2).

## Discussion

We conclude that the SNPs rs1800693 (*TNFRSF1A*) and rs17445836 (*IRF8*) are convincingly associated to MS in this independent replication set. This supports the role of these genes in MS etiology. The rs17824933 (*CD6*) showed nominally significant association in the analysis combining the replication cohorts, although the association in most of the individual cohorts was not significant. It is possible that the lack of association in some cohorts is due to true population heterogeneity, but the individual cohorts in our study do not have enough power to draw any definite conclusions. Especially, since the cohorts showing an opposite trend have little power by themselves. None of these three genes (*CD6*, *TNFRSF1A* or *IRF8*) had shown association above the replication inclusion threshold in the IMSGC [2] or Gene MSA [16] original publications (p<10^−4^), but by combining the data in a meta-analysis the full advantage of these cohorts could be used to mine more MS susceptibility affecting genes [11].

Rare mutations in previously validated MS susceptibility genes have been implicated in rare monogenic disorders. For example, mutations in *IL2RA* [17] and *IL7R* [18] cause immunodeficiency and mutation in *TYK2* [19] and *STAT3* [20] have been reported to cause hyper-IgE syndrome. Similarly, mutations in *TNFRSF1A* can cause TRAPS, a disease of the immune system characterized by periodic fevers [21]. It is interesting, that both TRAPS and relapsing-remitting form of multiple sclerosis are characterized by periodic activations of autoimmunity. A recent study in a small German cohort reported that 24% (6/25) of patients with clinically isolated syndrome (CIS) or MS with TRAPS-like symptoms were carrying an amino-acid changing allele R92Q of the SNP rs4149584 in *TNFRSF1A* [22]. In addition, they reported that the frequency of the R92Q allele was 4.66% in a general MS patient sample set (n 365) and 2.95% in a population sample (n 407) (p 0.112) [22].

*TNFRSF1A* codes for the precursor of TNF binding protein 1 and TNFR superfamily member 1A, a receptor that binds TNF-alpha and -beta, is involved in inflammatory responses and mediates apoptosis [23]. Experiments using knockout mice have shown, that mice with no functional p55 (TNFR1/Tnfrsf1a/CD120a) receptor were resistant to experimental autoimmune encephalomyelitis (EAE), the rodent model of MS [24]. On the other hand, clinical studies using lenercept, a recombinant TNF receptor p55 immunoglobulin fusion protein (sTNFR-IgG p55) that protects against EAE, reported increased exacerbation in a phase I safety trial patients using lenercept compared to patients using placebo [25].

CD6 is a T cell surface antigen involved in cell-cell adhesion [26]. It shares a role with a previously identified MS associated gene CD58 [3] in affecting the adhesion of the immune cells [27]. Interestingly, CD6 has been suggested to play a role in the apoptosis-resistance and positive selection of immature thymocytes during their maturation in thymus [28]. IRF8 is an interferon sensitive response element (ISRE) binding transcription factor expressed in cells of the immune system and responding to type 1 interferon stimulus [29]. It has been reported to regulate macrophage differentiation [30], has a critical role in the development of myeloid cells [31] and is likely involved in B-cell lineage specification, commitment and differentiation [32]. Both CD6 and IRF8 are involved in the development and maturation of leukocytes, which seems to emphasize the assumed autoimmune nature of MS.

*TNFRSF1A*, *IRF8* and *CD6* fit into the gradually emerging picture of the MS etiology as they have functions in various pathways involved in regulation of inflammatory responses in adaptive immunity and development of the immune system together with the previously identified MS associated genes *HLA-DRB1* [1], *IL7R* [2], *IL2RA* [2], *CLEC16A* [2], [4] and *CD58* [3], *TYK2* [5], *STAT3* [6] [6], *IL12A*, *MPHOSPH9/CDJ2AP1*, *KIF21B* [2], [7], *TMEM39A* [2], [7], *CYP27B1* [8], *CD226* [4], *CD40* [8], *CBLB* [9] and *RGS1* [10]. Thus, detailed fine mapping of these three genes together with other previously identified loci is needed to identify the causative variants. Future functional characterization of the identified variants will refine their role in MS pathogenesis and will enable the search for potential pathways and targets for future interventions.

## Materials and Methods

### Ethics Statement

All patient samples were collected with written informed consent. The study has been approved by appropriate local ethics committees: for Finnish sample collection and study design the Helsinki University Hospital ethics committee of ophthalmology, otorhinolaryngology, neurology and neurosurgery (permit no. 192/E9/02), for the Belgian cohort Commissie voor medische ethiek/klinisch onderzoek, Faculteit Geneeskunde K.U.Leuven (permit ML4733), for the Danish cohort The Danish Research Ethics Committee (permit KF 01314 009). The ethics committee approvals for all cohorts are listed in Table S3.

### Samples and genotyping

All samples had clinically definite MS by either the Poser criteria or McDonald criteria and anonymous population samples from respective populations were used as controls. (Table 1) All cohorts used in this independent replication were genotyped in local centers using either Taqman (Applied Biosystems, CA,USA) or Sequenom® iPLEX® Gold platform (SEQUENOM, CA, US) and manufacturer protocols, except for the Danish and Norwegian samples that were genotyped in Finland for rs17445836 and rs17824933 (Sequenom® iPLEX® Gold) (Table 1). The original meta-analysis sample sets from De Jager et al., that we used in the combined analysis of the original GWA and our replication results (Figure 1, last line), and their genotyping have been described elsewhere [11], [16].

### Statistical analyses

We excluded from the analysis all samples with >1 missing genotype and SNPs with <90% success rate or Hardy-Weinberg disequilibrium (HWE) p<0.001 per population. Using these criteria we excluded rs17445836 (*IRF8*) from the Spanish and German cohorts and rs1800693 (*TNFRSF1A*) from the Danish and French cohorts.

We performed both an independent replication analysis and a combined analysis using the original De Jager et al. GWA sample set. The analyses were performed according to Kazeem and Farral [15] and the calculations were done using R 2.9.0 (www.r-project.org). The Hardy-Weinberg (dis)equilibrium analysis p values were calculated using PLINK v1.06 (http://pngu.mgh.harvard.edu/~purcell/plink/). The T (Transmitted alleles) and U (Undertransmitted alleles) for the case-parent trios have been obtained from PLINK v1.06 transmission disequilibrium test (TDT) analysis.

## Supporting Information

Table S1 **Power calculations for all study sets.** All calculations were done using Researcher's toolkit's Statistical Power Calculator's two-tailed test with percentages by DSS (http://www.dssresearch.com/toolkit/spcalc/power_p2.asp) alpha = 5% for false positive probability, fixed MAFs calculated from the ORs of the combined effects and allele frequencies from the original study by De Jager et al. 2009. These results show that most of the individual sample sets have only moderate power to detect the association by themselves, but together have over 99% power to detect these variants with these effect sizes. The power for trios was not estimated.
(DOC)

Table S2 **Differences in rs17824933, rs1800693 and rs17445836 minor allele frequencies between population based controls.** This table shows results for pair-wise associations between controls from different populations. We used the controls from populations on the left as cases and controls from the population above as controls. For French samples, healthy parents from case-parent trio samples were used as population controls. Uncorrected p-values are shown, but all values below p 0.000303 are significant (α = 0.05) after Bonferroni correction. Table S2a has the results for rs17624933 in *CD6*, Table S2b describes the results for rs1800693 in *TNFRSF1A* and Table S2c describes results for 17445836 61.5 kb from *IRF8*.
(DOC)

Table S3 **Ethics committee approvals for all cohorts.** This study has been approved by appropriate local ethics committees as listed in this table by sample set. For each cohort we report the ethics committee or equivalent authority and the approval number.
(DOC)
