# Supplementary material for: The Genetic Association of Variants in CD6, TNFRSF1A and IRF8 to Multiple Sclerosis: A Multicenter Case-Control Study
Source: PLoS One. 2011 Apr 28;6(4):e18813. doi: 10.1371/journal.pone.0018813 (PMC3084233; doi:10.1371/journal.pone.0018813)
Supplement: Table S1 — Power calculations for all study sets. All calculations were done using Researcher's toolkit's Statistical Power Calculator's two-tailed test with percentages by DSS (http://www.dssresearch.com/toolkit/spcalc/power_p2.asp) alpha = 5% for false positive probability, fixed MAFs calculated from the ORs of the combined effects and allele frequencies from the original study by De Jager et al. 2009. These results show that most of the individual sample sets have only moderate power to detect the association by themselves, but together have over 99% power to detect these variants with these effect sizes. The power for trios was not estimated. (DOC) [file pone.0018813.s001.doc]

**Table S1. Power calculations for all study sets**

|  |  |  |  | **Power with alpha =5%** | | |
| --- | --- | --- | --- | --- | --- | --- |
|  |  |  |  | **CD6** | **TNFRSF1A** | **IRF8** |
|  | **N** | |  | **OR 1.18** | **OR 1.2** | **OR 0.80** |
| **Sets** | **MS cases** | **controls** | **N trios** | **MS 0.263, ctrl 0.232** | **MS 0.434, ctrl 0.390** | **MS 0.184, ctrl 0.220** |
| Belgium (BE) | 776 | 1021 | 0 | 32.8 | 46.8 | 46.6 |
| Denmark (DK) | 634 | 1090 | 0 | 30.6 | 43.4 | 42.7 |
| Finland (FI) | 792 | 1077 | 0 | 33.8 | 48.1 | 47.8 |
| France (FR) | 0 | 0 | 608 | n.a. | n.a. | n.a. |
| Germany (DE) | 930 | 911 | 0 | 33.8 | 48.3 | 48.6 |
| Italy (IT) | 828 | 629 | 0 | 27.1 | 39.3 | 40.0 |
| Norway (NO) | 662 | 1027 | 0 | 30.6 | 43.5 | 43.0 |
| Spain (ES) | 501 | 501 | 0 | 20.6 | 29.3 | 29.4 |
| Sweden (SE) | 2016 | 1723 | 0 | 59.0 | 77.8 | 78.1 |
| United Kingdom (UK) | 656 | 714 |  | 26.5 | 38.0 | 38.0 |
| United States (US) | 644 | 587 | 0 | 24.1 | 34.7 | 35.0 |
| *Total* | *8439* | *9280* | *608* | *99.8* | *100* | *100* |
