# Supplementary material for: The Genetic Association of Variants in CD6, TNFRSF1A and IRF8 to Multiple Sclerosis: A Multicenter Case-Control Study
Source: PLoS One. 2011 Apr 28;6(4):e18813. doi: 10.1371/journal.pone.0018813 (PMC3084233; doi:10.1371/journal.pone.0018813)
Supplement: Table S2 — Differences in rs17824933, rs1800693 and rs17445836 minor allele frequencies between population based controls. This table shows results for pair-wise associations between controls from different populations. We used the controls from populations on the left as cases and controls from the population above as controls. For French samples, healthy parents from case-parent trio samples were used as population controls. Uncorrected p-values are shown, but all values below p 0.000303 are significant (α = 0.05) after Bonferroni correction. Table S2a has the results for rs17624933 in CD6, Table S2b describes the results for rs1800693 in TNFRSF1A and Table S2c describes results for 17445836 61.5 kb from IRF8. (DOC) [file pone.0018813.s002.doc]

**Table S2a. Differences in rs17824933 (*CD6*) minor allele frequency between population based controls**

| *Population* | **Belgium** | **Denmark** | **Finland** | **France** | **Germany** | **Italy** | **Norway** | **Spain** | **Sweden** | **United Kingdom** |
| --- | --- | --- | --- | --- | --- | --- | --- | --- | --- | --- |
| **Denmark** | 0.246 | – |  |  |  |  |  |  |  |  |
| **Finland** | 0.00460 | 5.05x10^-5^ | – |  |  |  |  |  |  |  |
| **France** | 6.68x10^-7^ | 0.000103 | 2.31x10^-15^ | – |  |  |  |  |  |  |
| **Germany** | 0.00144 | 0.0348 | 2.07x10^-9^ | 0.116 | – |  |  |  |  |  |
| **Italy** | 4.18x10^-7^ | 4.06x10^-5^ | 1.75x10^-14^ | 0.425 | 0.0349 | – |  |  |  |  |
| **Norway** | 0.5427 | 0.490 | 1.75x10^-14^ | 4.64x10^-5^ | 0.0149 | 1.75x10^-5^ | – |  |  |  |
| **Spain** | 2.86x10^-6^ | 0.000146 | 1.57x10^-12^ | 0.411 | 0.0445 | 0.919 | 6.08x10^-5^ | – |  |  |
| **Sweden** | 0.562 | 0.0567 | 0.0101 | 2.90x10^-10^ | 3.32x10^-5^ | 1.01x10^-9^ | 0.222 | 3.11x10^-8^ | – |  |
| **United Kingdom** | 0.0183 | 0.182 | 5.65x10^-7^ | 0.0358 | 0.540 | 0.0106 | 0.0899 | 0.015 | 0.00171 | – |
| **United States** | 0.00344 | 0.0502 | 5.04x10^-8^ | 0.203 | 0.926 | 0.0701 | 0.0230 | 0.0783 | 0.000216 | 0.523 |

**Table S2b. Differences in rs1800693 (*TNFRSF1A*) minor allele frequency between population based controls**

| *Population* | **Belgium** | **Denmark** | **Finland** | **France** | **Germany** | **Italy** | **Norway** | **Spain** | **Sweden** | **United Kingdom** |
| --- | --- | --- | --- | --- | --- | --- | --- | --- | --- | --- |
| **Denmark** | na | – |  |  |  |  |  |  |  |  |
| **Finland** | 0.0334 | na | – |  |  |  |  |  |  |  |
| **France** | na | na | na | – |  |  |  |  |  |  |
| **Germany** | 0.135 | na | 0.573 | na | – |  |  |  |  |  |
| **Italy** | 0.103 | na | 0.838 | na | 0.773 | – |  |  |  |  |
| **Norway** | 0.000938 | na | 9.35x10^-8^ | na | 3.31x10^-6^ | 7.20x10^-6^ | – |  |  |  |
| **Spain** | 0.0792 | na | 0.000645 | na | 0.00370 | 0.00328 | 0.342 | – |  |  |
| **Sweden** | 0.87 | na | 0.0258 | na | 0.131 | 0.102 | 0.000145 | 0.0460 | – |  |
| **United Kingdom** | 0.293 | na | 0.381 | na | 0.734 | 0.561 | 5.03x10^-5^ | 0.0123 | 0.313 | – |
| **United States** | 0.0620 | na | 0.956 | na | 0.591 | 0.818 | 2.79x10^-6^ | 0.00176 | 0.0586 | 0.415 |

**Table S2c. Differences in rs17445836 (*IRF8*) minor allele frequency between population based controls**

| *Population* | **Belgium** | **Denmark** | **Finland** | **France** | **Germany** | **Italy** | **Norway** | **Spain** | **Sweden** | **United Kingdom** |
| --- | --- | --- | --- | --- | --- | --- | --- | --- | --- | --- |
| **Denmark** | 0.0669 | – |  |  |  |  |  |  |  |  |
| **Finland** | 2.14x10^-8^ | 0.000110 | – |  |  |  |  |  |  |  |
| **France** | 0.00523 | 1.96x10^-6^ | 4.07x10^-18^ | – |  |  |  |  |  |  |
| **Germany** | na | na | na | na | – |  |  |  |  |  |
| **Italy** | 1.89x10^-9^ | 3.04x10^-14^ | 1.28x10^-26^ | 0.000126 | na | – |  |  |  |  |
| **Norway** | 0.542 | 0.255 | 2.84x10^-6^ | 0.00101 | na | 2.29x10^-10^ | – |  |  |  |
| **Spain** | na | na | na | na | na | na | na | – |  |  |
| **Sweden** | 0.04198 | 0.976 | 2.07x10^-5^ | 1.55x10^-7^ | na | 9.43x10^-16^ | 0.215 | na | – |  |
| **United Kingdom** | 0.402 | 0.0121 | 3.72x10^-9^ | 0.0940 | na | 7.90x10^-7^ | 0.173 | na | 0.00652 | – |
| **United States** | 0.0691 | 0.000710 | 6.83x10^-11^ | 0.619 | na | 0.000142 | 0.0226 | na | 0.000296 | 0.332 |
