# Supplementary material for: The Genetic Association of Variants in CD6, TNFRSF1A and IRF8 to Multiple Sclerosis: A Multicenter Case-Control Study
Source: PLoS One. 2011 Apr 28;6(4):e18813. doi: 10.1371/journal.pone.0018813 (PMC3084233; doi:10.1371/journal.pone.0018813)
Supplement: Table S3 — Ethics committee approvals for all cohorts. This study has been approved by appropriate local ethics committees as listed in this table by sample set. For each cohort we report the ethics committee or equivalent authority and the approval number. (DOC) [file pone.0018813.s003.doc]

**Table S**3. Ethics committee approvals for all cohorts.

| **Sets** | **Ethics committee / Authority** | **Approval** |
| --- | --- | --- |
| Belgium (BE) | Commissie voor medische ethiek/klinisch onderzoek, Faculteit Geneeskunde K.U.Leuven | ML4733 |
| Denmark (DK) | The Danish Research Ethics Committee | KF 01314 009 |
| Finland (FI) | Helsinki University Hospital ethics committee of ophthalmology, otorhinolaryngology, neurology and neurosurgery | 192/E9/02 |
| France (FR) | The French Ministry of research | DC-2008-539  AC-2008-548 |
| Germany (DE) | Ethics committees of the universities of Marburg, Duesseldorf and Munich | 1856/07 |
| Italy (IT) | Comitato Etico (CE) Interaziendale, Novara, Italy | 570/CE, N. CE 38/0 |
| Norway (NO) | Ethical Committee South Eastern Norway  Local Data Inspectorate at Oslo University Hospital | S-08234a  2476 |
| Spain (ES) | Institut Català, Hospital Vall d’Hebron, Comité Ètic | PR(AG)30/2007 |
| Sweden (SE) | Regionala Etikprövningsnämnde, Stockholm, Sweden  Karolinska Institutets Regionala Forskningskomitté | 04-252/1-4; 2006/845-31/1  00-052, 04-375; 02-548 |
| United Kingdom (UK) and | Partners Healthcare Institutional Review Board | 2002-p-000434 |
| United States (US) |  |  |
